# Supplementary material for: Vancomycin Area under the Concentration-Time Curve Estimation Using Bayesian Modeling versus First-Order Pharmacokinetic Equations: A Quasi-Experimental Study
Source: Antibiotics (Basel). 2022 Sep 13;11(9):1239. doi: 10.3390/antibiotics11091239 (PMC9495010; doi:10.3390/antibiotics11091239)
Supplement: Supplementary file 1 [file antibiotics-11-01239-s001.zip › antibiotics-1914680-supplementary.pdf]

Supplementary Table S1: Comparison of AUC estimations when 2 vancomycin concentrations were drawn in the same dosing interval

Patient	AUC per 2-Concentration PK	AUC_{SS} per Bayesian 2-Concentration	AUC_{SS} per Bayesian First Concentration	AUC_{SS} per Bayesian Second Concentration	Point AUC per Bayesian 2-Concentration*	Point AUC per Bayesian First Concentration*	Point AUC per Bayesian Second Concentration*	Bayesian Model
1a	405	503	560	526	354	348	456	Carreno
1b	669	720	706	718	700	690	686	Carreno
1c	570	539	459	579	588	469	585	Carreno
1d	531	506	526	485	526	578	503	Carreno
2	669	683	695	671	727	734	712	Thomson
3	451	465	490	465	462	480	452	Thomson
4	796	1041	1000	1062	782	762	809	Thomson
5	709	797	760	754	613	552	547	Thomson
6	553	537	547	538	537	563	559	Carreno
7a	**563**	**644**	582	589	**619**	**622**	499	Carreno
7b	426	484	507	487	517	555	520	Carreno
8	584	501	500	433	494	489	425	Thomson
9a	340	343	343	326	339	339	295	Carreno
9b	**535**	**616**	**638**	578	557	574	526	Carreno

Supplementary Table 1. Post hoc analysis: AUCs by different methods of estimation using the 14 AUC assessments from the 2-concentration cohort
*Point AUC is the estimated AUC at the time of the dose immediately prior to the concentration rather than the predicted steady state AUC
AUC=area under the concentration-time curve, AUC_{SS} = AUC at steady state, PK = pharmacokinetic

Supplementary Figure S1: Vancomycin AUC monitoring protocol for study staff

Vancomycin AUC Monitoring Protocol

<u>Overview</u>

1. All patients admitted to the 10th floor who receive IV vancomycin will be enrolled in the pilot study from 11/12/19 to 1/13/20 (10 weeks).

2. Study patients will be divided into 2 arms:
   a. In the first 5 weeks (11/12/19 to 12/16/19), patients on **10 A and B** will have their vancomycin AUC calculated with Bayesian software (InsightRX), while patients on **10 C and D** will have their vancomycin AUC calculated using first-order PK equations based on 2 serum vancomycin levels using the Epic PK navigator.
   b. After 5 weeks, the AUC measurement method will be switched for these pods (see table).

Pod:	11/12/19 to 12/16/19	12/17/19 to 1/13/20
10A	Bayesian	2-level
10B	Bayesian	2-level
10C	2-level	Bayesian
10D	2-level	Bayesian

<u>Vancomycin Initiation</u>

3. When a new vancomycin order is placed, the study pharmacist will open an i-Vent with information about the study protocol and the AUC calculation method. The following dotphrases **.vancobayesian**, **.vancoPK, or vancoFU** were created to standardize the i-Vent note. The i-Vent type should be filed as '**Antimicrobial Stewardship**' with the subtype '**Therapeutic Monitoring**' for consistency.

4. The covering study team pharmacist will also order or re-time appropriate vancomycin level(s) based on the AUC calculation method being used for the specific location.

   a. In the first-order PK equations 2-serum levels arm, patients should have **two** vancomycin levels drawn on the same dosing interval after the 3rd or 4th dose; the first level should be timed for **4 hours after** the documented MAR administration time of the dose and and the second one timed for **1 hour before** the next dose is scheduled in the MAR. If the patient has received several doses of vancomycin and has had levels taken, another trough level should be ordered 3 days after the last level taken if the renal function has remained stable.

   b. In the Bayesian arm, **a single** vancomycin level should be ordered to be drawn **with morning labs**, unless a vancomycin dose is scheduled to be given between 2-6 AM. If vancomycin is scheduled to be administered between 2-6 AM, then a **single** vancomycin level should be timed for 1 hour before the next dose. If the

patient has received several doses of vancomycin and has had levels taken, another trough level should be ordered 3 days after the last level taken if the renal function has remained stable.

5. Patients should be entered into the REDCap Database at the time of vancomycin initiation and the "Initial Assessment" portion of REDCap filled out.
6. Patient information should be filled out in the shared Dropbox folder for the daily email

Vancomycin Follow-Up

7. When Theradoc emails the study team with a **vancomycin level result** (not vancomycin order), the covering study team member will open Epic to the patient chart and then start the timer in Toggl, then calculate the patient's AUC. If the AUC is between 400-600 and the trough is >10 mg/L, no change will be made to the current regimen. If the AUC is <400 or >600 OR the trough is <10 mg/L, a new dosing regimen targeting an AUC of ~500 with a trough of >10 mg/L will be recommended.
    a. In the first-order PK equations 2-serum levels arm, the kinetics navigator in Epic will be used, using the vancomycin tab → result assessment → 2-levels. The Toggl timer should collect from when the Epic patient chart is opened until the i-Vent is completed using the project "PK Navigator." A standard administration time of 120 minutes should be used for vancomycin doses ≤1.25 grams and 180 minutes for doses ≥1.5 grams.
    b. In the Bayesian arm, two activities will be timed and recorded in Toggl, the first being the time from obtaining the level to inputting all new data into InsightRx under the project "Bayesian Patient Data." All doses administered, serum creatinines, and levels from the past 7 days should be entered into InsightRx with patient name entered as "Patient" as First Name and REDCap ID as Last Name and MRN (CSN/ visit ID can be left blank). The birth date inputted in InsightRx should be at least 1 day, but no more than one month, off of the patient's actual date of birth. A standard administration time of 120 minutes should be used for vancomycin doses ≤1.25 grams and 180 minutes for doses ≥1.5 grams.
        i. Before clicking the update button in InsightRx, the "Bayesian Patient Data" timer should be stopped in Toggl and switched to "Bayesian Assessment." Then the time from InsightRx results to the time to document dose adjustments and update the i-Vent will be recorded. This is to simulate the time it would take to estimate an AUC and make vancomycin dose adjustements if InsightRx was integrated in the electronic health record. In InsightRx, the Thomson model will be used for most patients while the Carreno (obese) model used when InsightRx recommends it for obese patients.

8. Once the current regimen is either determined to be appropriate or a new regimen is determined, the study team will update the i-Vent and then stop the Toggl timer. Please

ensure that the Toggl timer is appropriately tagged to the correct project and that the patient's REDCap ID is in the description.

9. The study team will add any patient levels to the REDCap database under the "Vancomycin Level Assessment" instrument.

10. Patient information should be updated in the shared Dropbox folder for the daily email.

11. The study team will FYI page the covering pharmacist with the vancomycin dosing recommendations, even if the dose is unchanged. Additionally, the study team will page the responding clinician with recommendations for the AUC-calculated vancomycin regimen, even if dose is unchanged.

12. The study team will continually monitor patients while they are receiving IV vancomycin therapy on the 10th floor. The study team will update the floor pharmacists and the responding clinicians upon any new levels. Patients that remain on the 10th floor on vancomycin for more than 5 days or with a change in SCr of ≥0.5 mg/dL may have vancomycin levels redrawn to reassess the regimen. If a dose change is made, levels can be -rechecked in 3 days.

13. A nightly email with all active vancomycin patients will be sent out to the study team and staff pharmacists covering the the 10th floor.

Supplementary Figure S2: Example education – pharmacist and physician presentation

BRIGHAM HEALTH

BWH BRIGHAM AND WOMEN'S HOSPITAL

Vancomycin AUC Monitoring Pilot

Jeffrey Pearson, PharmD, BCIDP
Senior Pharmacist, Infectious Diseases

November 13, 2019

HARVARD MEDICAL SCHOOL TEACHING HOSPITAL

MAGNET RECOGNIZED
AMERICAN NURSES CREDENTIALING CENTER

Vancomycin Toxicity

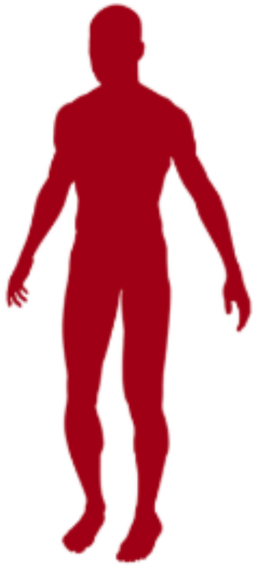

- Red-Man Syndrome
  - Infusion-related
  - Solution: slow down infusion, can potentially give antihistamines
- Nephrotoxicity
  - Dose-related
  - Solution(ish): therapeutic drug monitoring (TDM)
- Ototoxicity
  - Majority of data was published when less pure formulations were available

Nephrotoxicity Risk Factors

- Concomitant nephrotoxic agents
- Elderly
- Chronic kidney disease
- Critical illness
- Duration >7 days
- **Increased vancomycin troughs**
- **Increased vancomycin AUC**
- Vancomycin doses >4 grams/day

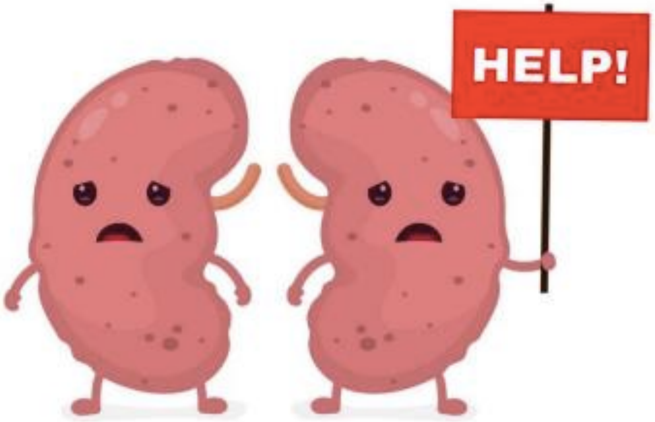

https://med.uth.edu/internalmedicine/files/2018/07/TEAKELL-AKI-2018.pdf

Vancomycin PK/PD

- Activity based on AUC:MIC ratio
- Troughs have been used as a surrogate marker for AUC

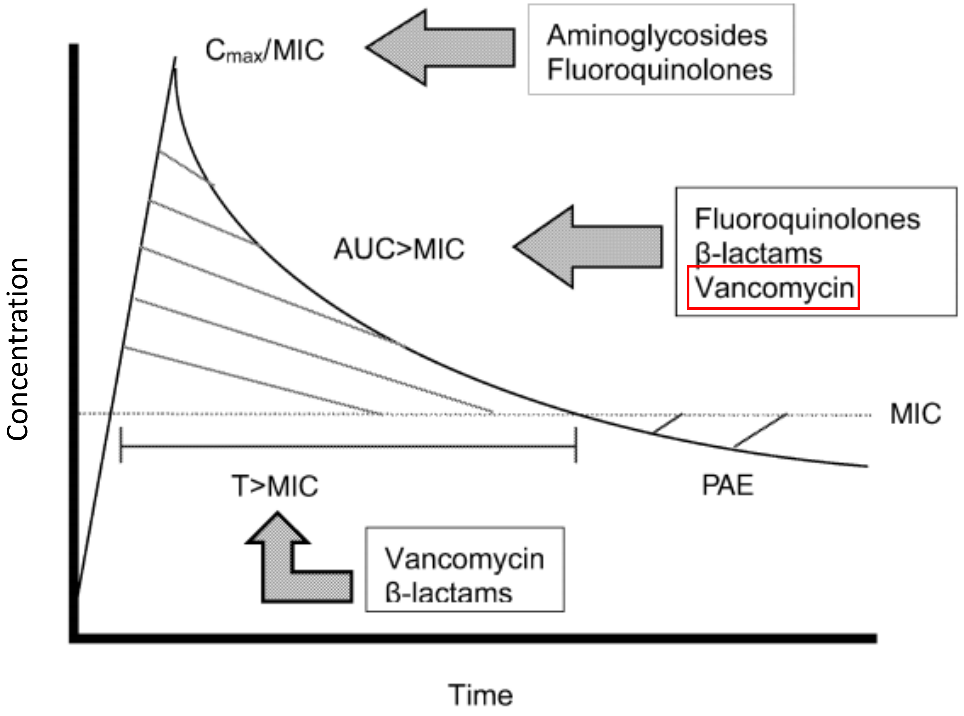

McKinnon PS, Davis SL. Eur J Clin Microbiol Infect Dis. 2004;23:271-288

Vancomycin PK/PD

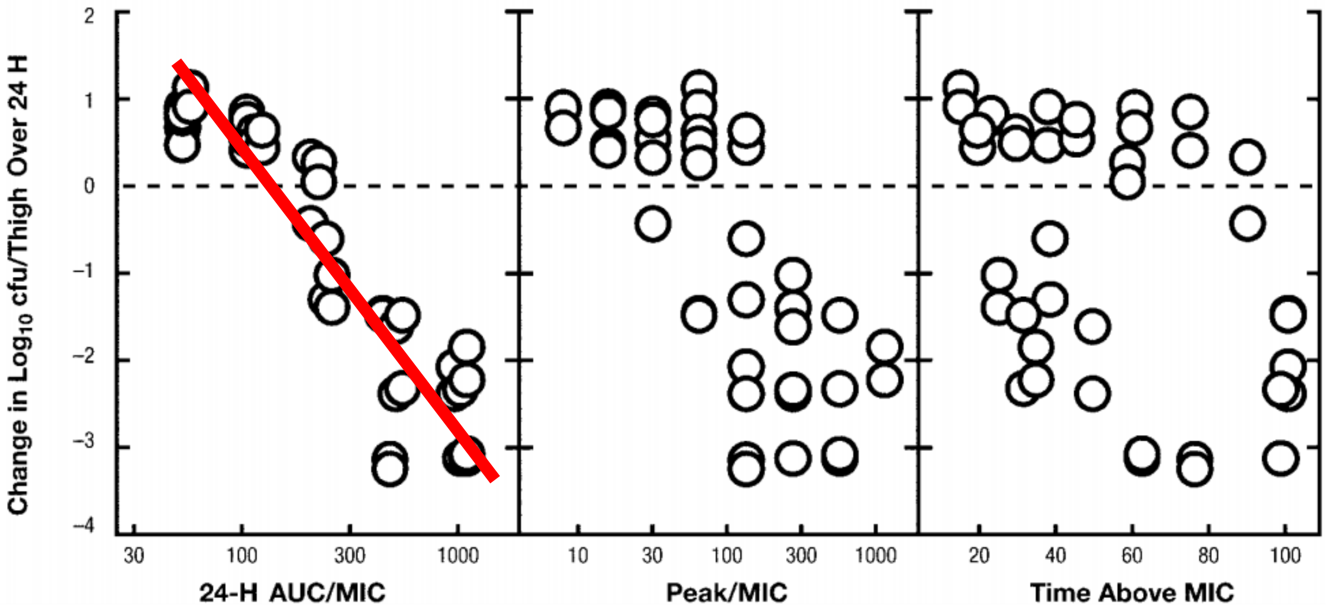

Figure 2. Relationship between pharmacokinetic/pharmacodynamic indices for vancomycin and bacteriologic efficacy against methicillin-susceptible *Staphylococcus aureus*. This plot, which delineates the change in colony-forming units (cfu) in an experimental mouse infection model 3 different ways, suggests that the area under the curve divided by the MIC (AUC/MIC) is the most valuable pharmacokinetic/pharmacodynamic parameter for predicting the activity of vancomycin against methicillin-susceptible *S. aureus*. Peak/MIC, peak serum concentration divided by the MIC. Data are from Ebert [23].

Rybak MJ. Clin Infect Dis. 2006;42:S35-39

Vancomycin PK/PD

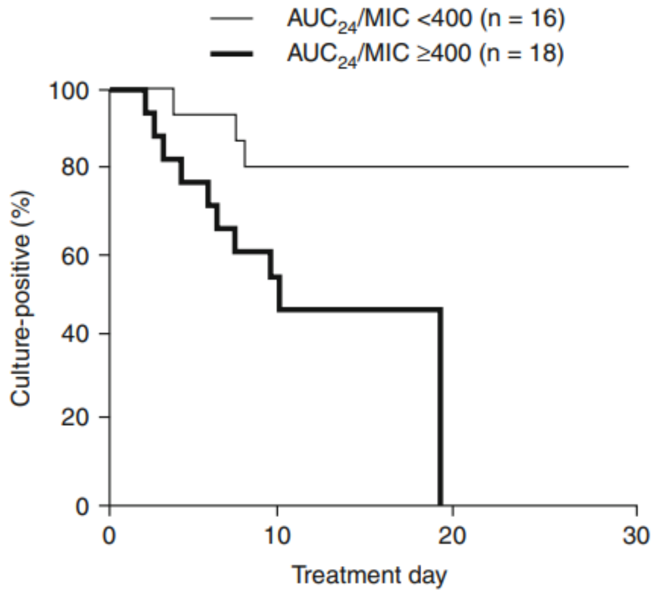

Fig. 4. Time (days of therapy) to bacterial eradication vs vancomycin AUC_{24}/MIC <400 and AUC_{24}/MIC ≥400 illustrated by a Kaplan-Meier survival plot of day of therapy vs the percentage of patients remaining culture-positive on that day. The two AUC_{24}/MIC groups differed significantly ($p = 0.0402$). **AUC_{24}/MIC** = steady-state 24-hour area under the concentration-time curve divided by the minimum inhibitory concentration.

Moise-Broder PA et al. Clin Pharmacokinet. 2004;43(13):925-942

Vancomycin AUC

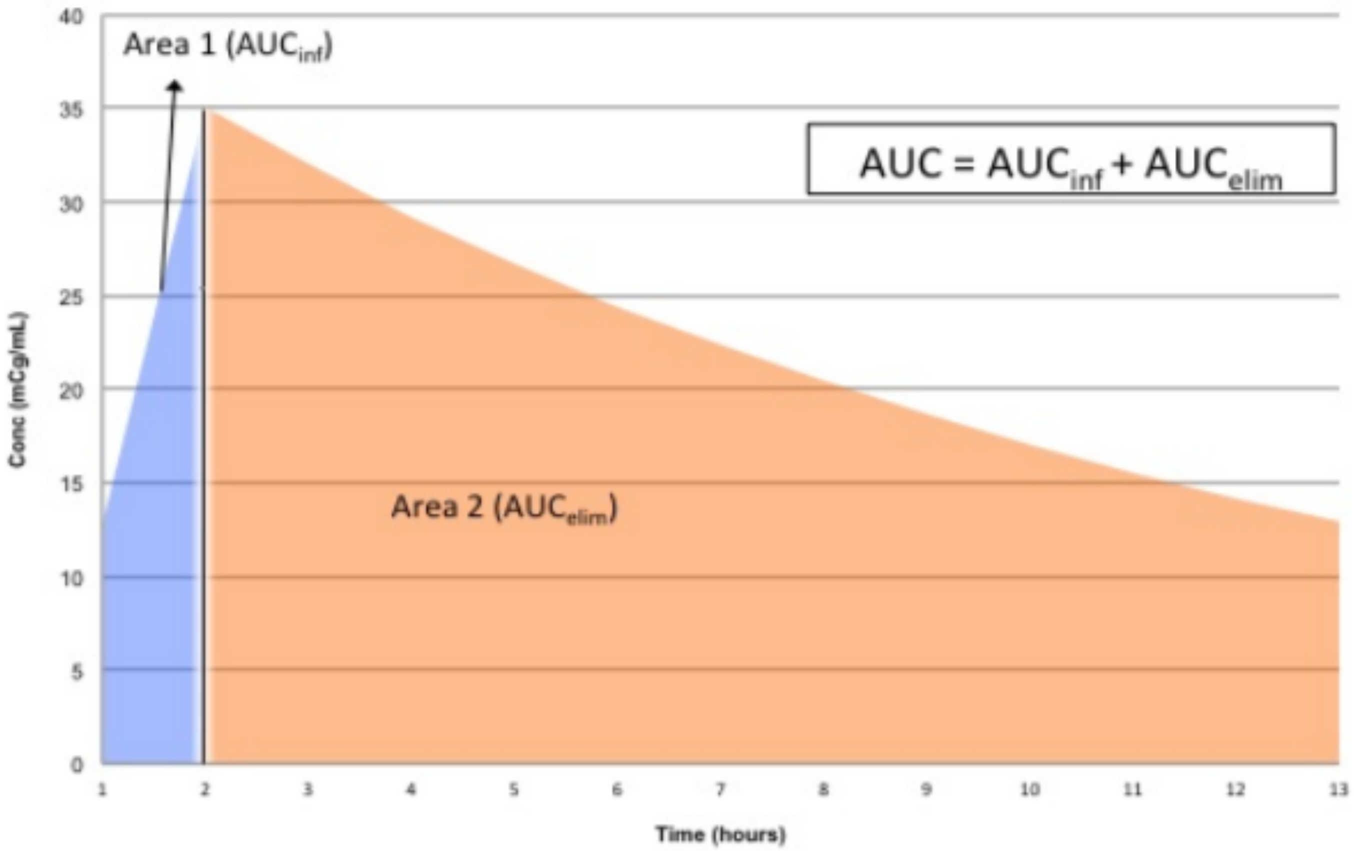

Courtesy of Detroit Medical Center

Vancomycin AUC

AUC during elimination [AUC_{elim}]

$$AUC_{elim} = \frac{C_{true\ peak} - C_{true\ trough}}{Kel}$$

Kel [Kel]

$$Kel = \left(\frac{\ldots}{(T_2 - T_1)} \right)$$

Predicted new C_{peak} [$C_{peak\ est}$]

$$C_{peak\ est} = \left(\frac{dose_{test}}{CL * T_{inf\ test}} \right) * \left(\frac{1 - e^{-Kel \times T_{inf\ test}}}{1 - e^{-Kel \times \tau_{test}}} \right)$$

[$C_{true\ trough}$]

$C_{true\ trough} = C_2 \ldots$

Predicted new C_{trough} [$C_{trough\ est}$]

$$C_{trough\ est} = C_{peak\ est} * e^{-Kel \times (\tau_{test} - T_{inf\ test})}$$

True Peak [C ...]

$C_{true\ pea\ldots}$

Predicted new AUC during infusion [$AUC_{inf\ test}$]

$$AUC_{inf\ test} = T_{inf\ test} * \frac{C_{peak\ est} + C_{trough\ est}}{2}$$

[$t_{1/\ldots}$]

$\ldots \ln 2 \ldots$

$\ldots_{1/2} \ldots Kel$

$AUC_{0\text{-}24}$ [$AUC_{0\text{-}24}$]

Predicted new AUC during elimination [$AUC_{elim\ test}$]

$$AUC_{elim\ test} = \frac{C_{peak\ est} - C_{trough\ est}}{Kel}$$

Vd calculated [Vd]

$$Vd\ (L) = \left[\frac{Dose \times (1 - e^{-Kel \times T_{inf}})}{Kel \times T_{inf}} \right] \times [Kel \times (\ldots$$

$$T_{inf} = \frac{\text{end user entered administer over time (min)}}{60}$$

Predicted new $AUC_{0\text{-}24}$ [$AUC_{0\text{-}24\ test}$]

$$AUC_{0-24\ test} = (AUC_{inf\ test} + AUC_{elim\ test}) * \frac{24}{\tau_{test}}$$

[$\ldots UC_{inf}$]

$$AUC_{inf} = T_{inf} * \frac{C_{true\ peak} + C_{true\ trough}}{2}$$

Vancomycin Trough as Surrogate

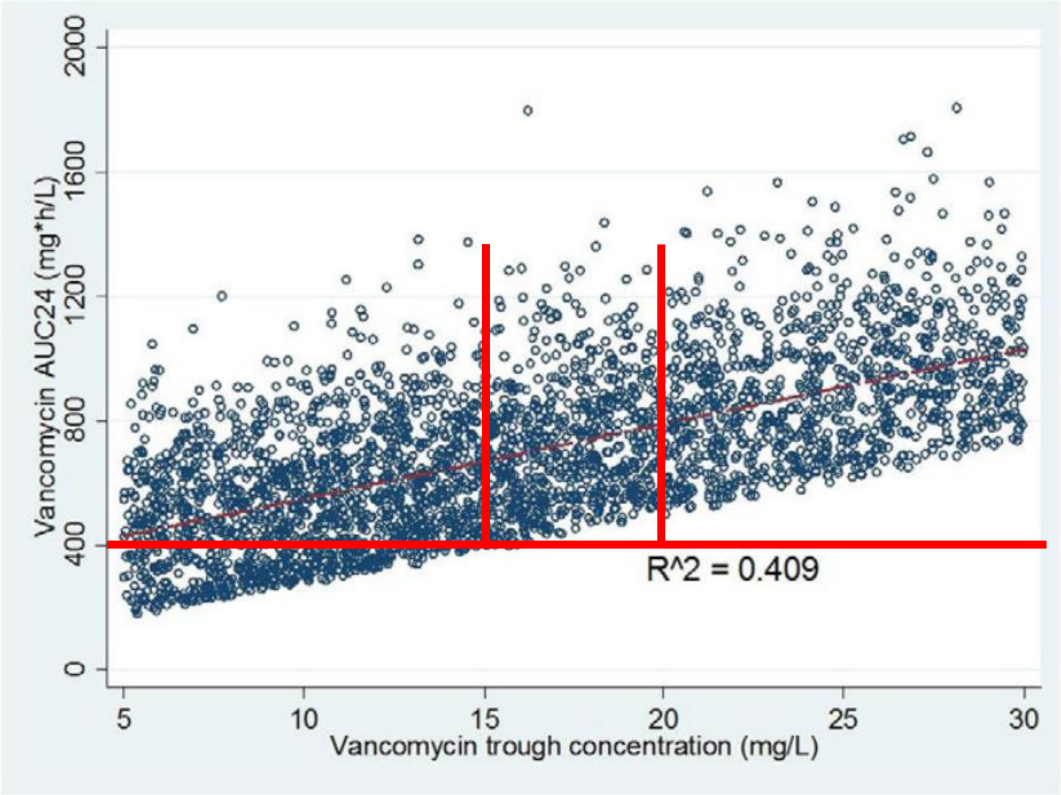

Fig. 2. Scatter and linear fit plot of vancomycin area under the curve over 24 h (AUC24) versus trough vancomycin concentration from 5000 subject Monte Carlo simulation.

Pai MP et al. Adv Drug Deliv Rev. 2014;77:50-57

Vancomycin Trough as Surrogate

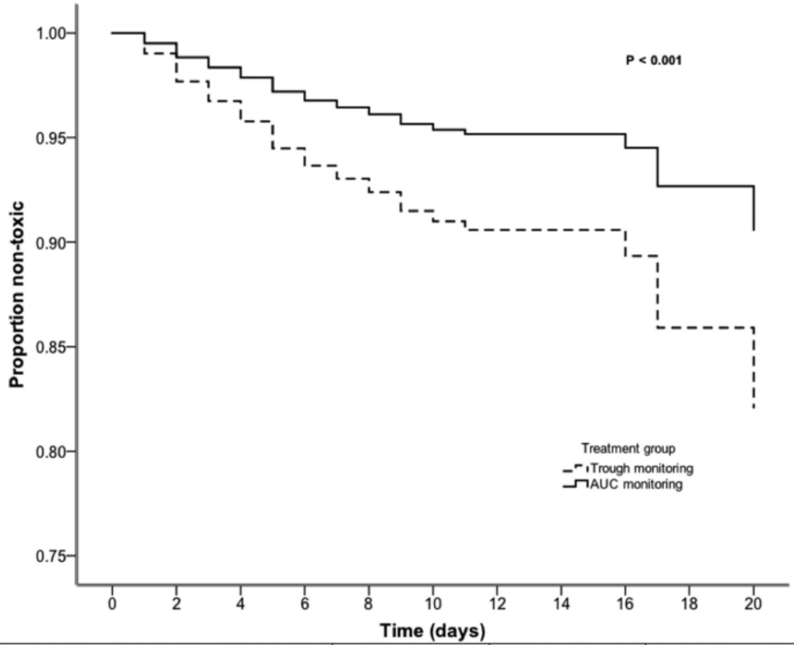

Variable	Hazard Ratio	95% CI	*P* value
AUC-TD	0.501	0.336 – 0.748	0.001
Concomitant furosemide	1.636	1.072 – 2.496	0.022
Elixhauser Comorbidity Index	1.123	1.044 – 1.208	0.002
APACHE II score	1.066	1.042 – 1.091	<0.001
Concomitant IV contrast	1.508	0.972 – 2.339	0.067
Concomitant tobramycin [a]	-	-	-
Duration of therapy, days [a]	-	-	-

[a] Not retained in final model

FIG 1 Time to nephrotoxicity by Cox proportional hazards regression. AUC-TD, AUC- and trough concentration-guided dosing.

Finch N et al. Antimicrob Agents Chemother. 2017;61(12):pii: e01293-17. doi: 10.1128/AAC.01293-17

Vancomycin Trough as Surrogate

Study or Subgroup	High troughs ≥15mg/L Events	High troughs ≥15mg/L Total	Low trough <15mg/L Events	Low trough <15mg/L Total	Weight	Odds Ratio M-H, Random, 95% CI
Bosso et al. (21)	42	142	13	146	9.8%	4.30 [2.19, 8.43]
Cano et al. (22)	22	89	7	99	7.2%	4.32 [1.74, 10.69]
Chung et al. (23)	12	25	16	48	6.5%	1.85 [0.69, 4.96]
Hermsen et al. (30)	5	16	4	39	3.6%	3.98 [0.91, 17.46]
Hidayat et al. (13)	11	63	0	32	1.1%	14.24 [0.81, 249.87]
Jeffres et al. (15)	27	49	13	45	7.7%	3.02 [1.28, 7.11]
Kralovicova et al. (31)	21	60	29	138	9.8%	2.02 [1.04, 3.96]
Kullar et al. (32)	8	116	1	84	2.0%	6.15 [0.75, 50.13]
Kullar et al. (8)	27	139	23	141	10.6%	1.24 [0.67, 2.28]
Lodise et al. (36)	7	27	14	139	6.2%	3.13 [1.12, 8.69]
McKamy et al. (38)	16	57	8	110	7.0%	4.98 [1.98, 12.52]
Minejima et al. (39)	17	72	25	155	9.6%	1.61 [0.80, 3.21]
Prabaker et al. (43)	7	54	24	294	7.3%	1.68 [0.68, 4.11]
Wunderink et al. (50)	26	118	24	215	10.7%	2.25 [1.22, 4.13]
Zimmermann et al. (51)	8	12	0	33	1.0%	126.56 [6.19, 2585.90]
**Total (95% CI)**		**1039**		**1718**	**100.0%**	**2.67 [1.95, 3.65]**
Total events	256		201			

Heterogeneity: $Tau^2 = 0.14$; $Chi^2 = 23.89$, $df = 14$ ($P = 0.05$); $I^2 = 41\%$
Test for overall effect: $Z = 6.13$ ($P < 0.00001$)

Odds Ratio
M-H, Random, 95% CI

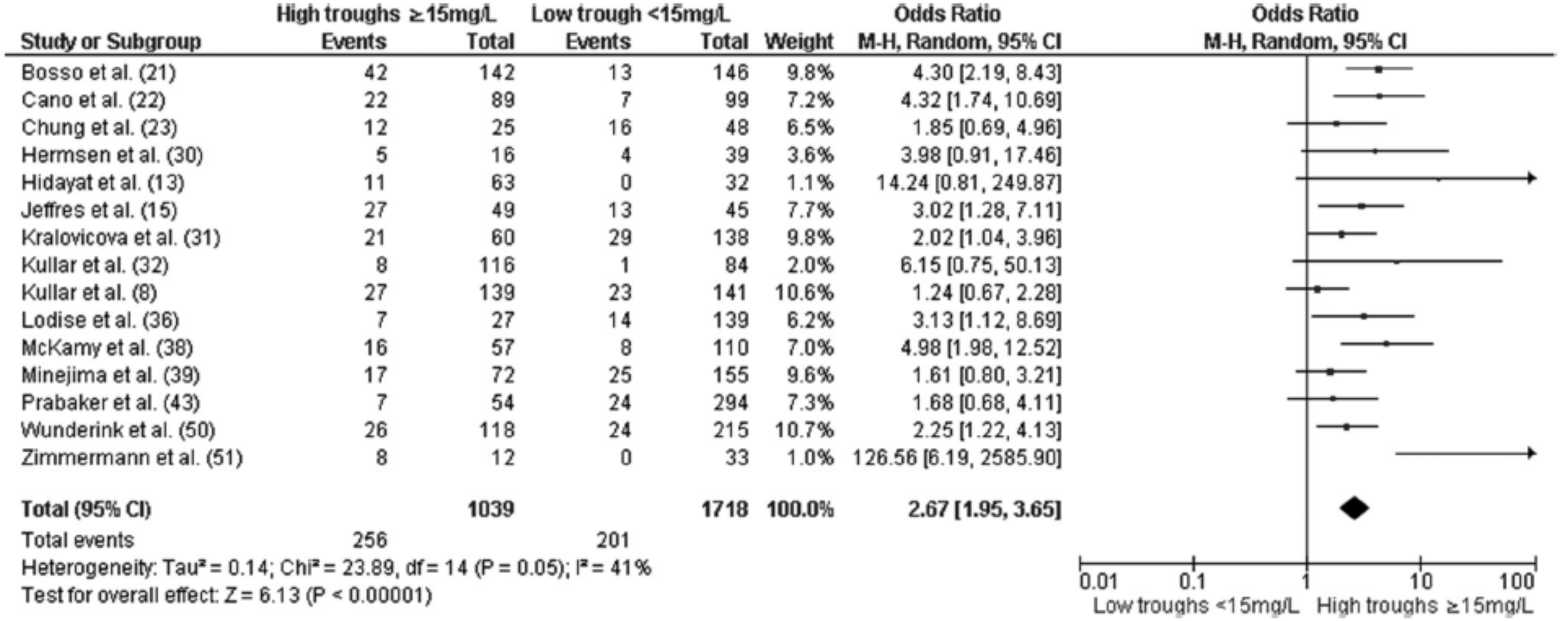

FIG 1 Forest plot (using Mantel-Haenszel [M-H] analysis) of events denoting nephrotoxicity associated with vancomycin, comparing rates for trough levels of ≥15 mg/dl and <15 mg/dl. Squares indicate point estimates, and the size of the square indicates the weight of each study.

van Hal SJ, et al. Antimicrob Agents Chemother. 2013 Feb;57(2):734-44

Vancomycin AUC Monitoring

Therapeutic monitoring of vancomycin: A revised consensus guideline and review of the American Society of Health-System Pharmacists, the Infectious Diseases Society of America, the Pediatric Infectious Diseases Society and the Society of Infectious Diseases Pharmacists

Rybak, MJ,[1-3] Le J,[4] Lodise, TP,[5,6] Levine DP,[2,3] Bradley, JS,[7,8] Liu, C,[9,10] Mueller, BA,[11] Pai, MP,[11] Wong-Beringer, A,[12] Rotschafer, JC,[13] Rodvold, KA,[14] Maples, HD,[15] Lomaestro, B.[16]

What does this mean for BWH?

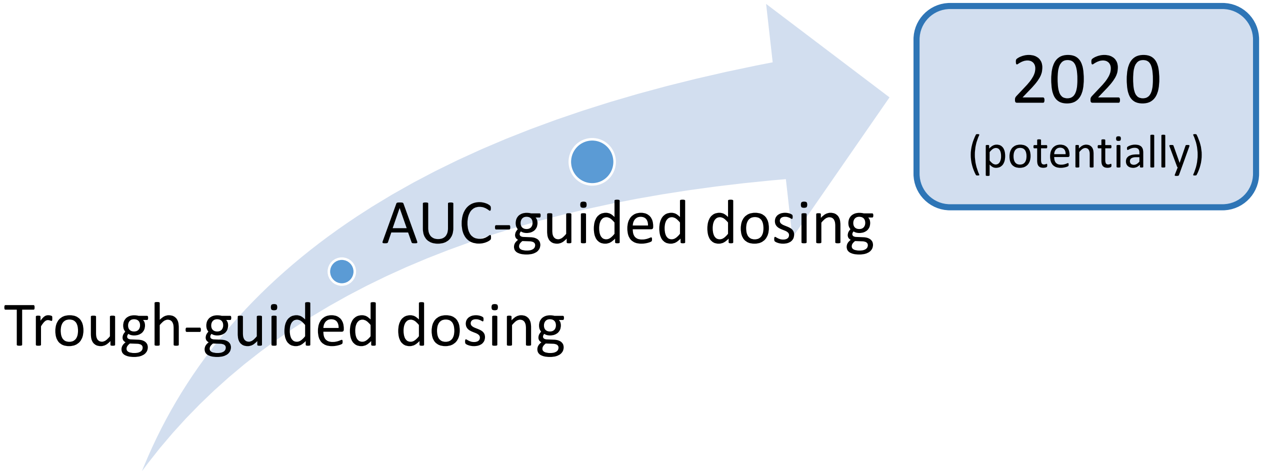

- Pilot study launched
  - 10th floor
  - November 12th to January 13th
  - Evaluates two different methods for estimating vancomycin AUC (goal 400-600)
    - This may result in therapeutic troughs that are <15 mcg/mL

What does this pilot mean for you?

- Study team will order or re-time vancomycin levels
  - To appropriately calculate vancomycin AUC
- Study team will page the responding clinician with any dose adjustments based on this calculation
- Level(s) drawn during the pilot should **not** be assessed as troughs and are purposefully ordered

What does this pilot mean for you?

Month 1

Two vancomycin levels will be drawn between vancomycin doses

- One random vancomycin level ~4 hours after administration & another as a trough

Month 2

One random vancomycin level with morning labs

Vancomycin AUC Pilot

- Questions?
- Email bwhidpharmacist@partners.org
- My pager is p34806
  - Or searchable in the directory as Jeffrey Pearson

BRIGHAM HEALTH

BWH BRIGHAM AND WOMEN'S HOSPITAL

Vancomycin AUC Monitoring Pilot

Jeffrey Pearson, PharmD, BCIDP
Senior Pharmacist, Infectious Diseases

November 13, 2019

HARVARD MEDICAL SCHOOL TEACHING HOSPITAL

MAGNET RECOGNIZED
AMERICAN NURSES CREDENTIALING CENTER

Supplementary Figure S3: Example education – nurse email and education on the floors

ATTENTION:
10 C/D NURSING & STAFF

VANCOMYCIN MONITORING PILOT

All adult patients receiving vancomycin and not on hemodialysis will be included in the pilot

November 12th - December 16th

Two vancomycin levels (both a peak and a trough level) will be ordered between vancomycin doses

If you have questions or concerns about a level, please contact the study team before canceling an ordered level or holding the vancomycin dose. Thank you!

QUESTIONS? FEEL FREE TO EMAIL THE STUDY TEAM AT BWHIDPHARMACIST@PARTNERS.ORG

Supplementary Figure S4: Example education – physician email

Pilot Study on Vancomycin AUC-Guided Dosing

Take-home points:

- BWH will be transitioning vancomycin monitoring from trough-only to area under the concentration-time curve (**AUC)-guided dosing** in 2020
- A pilot study is being conducted on the 10th floor from **November 12th to January 13th** to evaluate two different methods for estimating vancomycin AUC with a goal of 400-600. This may result in therapeutic troughs that are <15 mcg/mL
- During the study period, the **study team will be ordering or re-timing vancomycin serum levels** to appropriately calculate the vancomycin AUC and then contacting the responding clinician to adjust the vancomycin dose based on the calculated AUC
- Level(s) drawn during the pilot should not be assessed as troughs and are purposefully ordered
- **Please do not cancel these levels** without first checking with a member of the pharmacy study team via email at bwhidpharmacist@partners.org

Background:

Based on current literature, the vancomycin pharmacodynamic parameter most closely linked to efficacy is the ratio of the daily area under the concentration-time curve (AUC) to minimum inhibitory concentration (MIC), with a target AUC:MIC ratio of ≥400 for methicillin-resistant *Staphylococcus aureus*.[1] There are emerging consensus recommendations that will endorse AUC monitoring with a goal of 400-600 over trough-only monitoring.[2] Calculating vancomycin AUC can be performed either mathematically using first-order pharmacokinetic (PK) equations or with Bayesian modeling software.

Bayesian programs combine population estimates of vancomycin PK with patient-specific PK parameters and drug exposure data to predict optimal vancomycin dosing regimens.[2] There are several advantages with this method to estimate the vancomycin AUC: vancomycin serum concentrations can be assessed within the first 24 hours and they tend to be more accurate in patients who are obese and/or who have renal impairment.

Using first-order PK equations to calculate the AUC requires two vancomycin concentrations, preferably assessed once steady-state concentrations have been achieved.[2] A major limitation of this method is that it is not adaptive and only provides an estimation of the AUC during the sampling period. As such, it can be less accurate if a physiologic change such as renal dysfunction occurs during or after the sampling period, or if the patient has received different vancomycin doses in the previous 24 hours.

Our pilot study over the next two months will evaluate these two methods for estimating vancomycin AUCs, to assist in determining which method should be employed at BWH going forward. All patients on the 10th floor receiving vancomycin will be included in the study, with hemodialysis patients being excluded.

Contact bwhidpharmacist@partners.org with questions

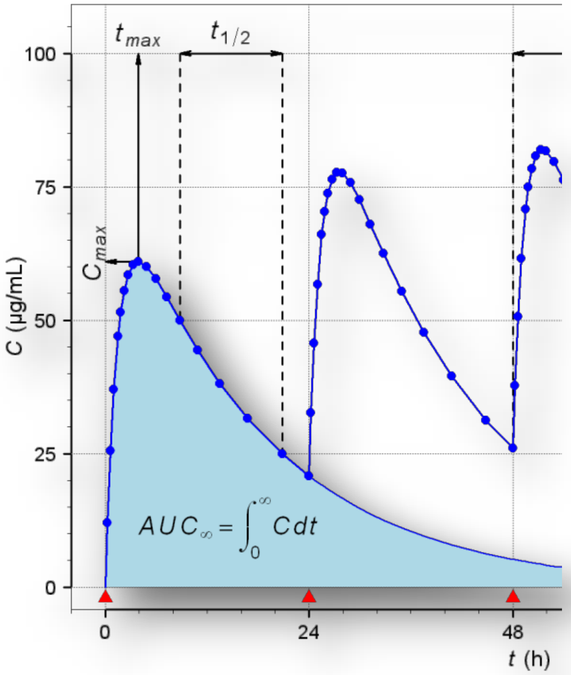

References:

1. Rybak MJ, Lomaestro B, Rotschafer JC et al. Therapeutic monitoring of vancomycin in adult patients: a consensus review of the American Society of Health-System Pharmacists, the Infectious Diseases Society of America, and the Society of Infectious Diseases Pharmacists. Am J Health Syst Pharm. 2009;66(1):82-98
2. Rybak MJ, Le J, Lodise TP et al. Therapeutic monitoring of vancomycin: A revised consensus guideline and review of the American Society of Health-System Pharmacists, the Infectious Diseases Society of America, the Pediatric Infectious Diseases Society and the Society of Infectious Diseases Pharmacists (Accessed November 7, 2019, at https://www.ashp.org/-/media/assets/policy-guidelines/docs/draft-guidelines/draft-guidelines-ASHP-IDSA-PIDS-SIDP-therapeutic-vancomycin.ashx)
